# Psychological Consequences of Social Isolation During COVID-19 Outbreak

**DOI:** 10.3389/fpsyg.2020.02201

**Published:** 2020-09-09

**Authors:** Giada Pietrabissa, Susan G. Simpson

**Affiliations:** ^1^Department of Psychology, Catholic University of Milan, Milan, Italy; ^2^Psychology Research Laboratory, IRCCS, Istituto Auxologico Italiano, Milan, Italy; ^3^Department of Justice and Society, University of South Australia, Adelaide, SA, Australia; ^4^Regional Eating Disorders Unit, NHS Lothian, Edinburgh, United Kingdom

**Keywords:** social isolation, COVID-19, psychological consequences, loneliness, depression, clinical psychology

## Abstract

Perceived social isolation during the COVID-19 pandemic significantly has had an extraordinary global impact, with significant psychological consequences. Changes in our daily lives, feeling of loneliness, job losses, financial difficulty, and grief over the death of loved ones have the potential to affect the mental health of many. In an atmosphere of uncertainty, it is essential that clear and precise information is offered about the problem and how to manage it. In this contribution, a rationale is provided for an urgent call for a rapid response to the mental health impacts of COVID-19. Moreover, suggestions for individuals to regulate their emotions effectively and appropriately are provided.

## Introduction

The mental health consequences of COVID-19 are already visible and even by conservative estimates they are yet to reach their peak and likely to considerably outlive the current pandemic.

The most common psychological disorders emerging are anxiety and panic, obsessive-compulsive symptoms, insomnia, digestive problems, as well as depressive symptoms and post-traumatic stress (Rogers et al., 2020). These are not only a direct consequence of the pandemic but also largely driven by the effects of prolonged social isolation – that is the objective lack of interactions with others (Leigh-Hunt et al., 2017). The medical journal *The Lancet* recently published an article from which a clear and alarming picture emerges: periods of isolation, even less than 10 days, can have long-term effects, with the presence – up to 3 years later – of psychiatric symptoms (Brooks et al., 2020).

Although necessary to limit the spread of the epidemic, in fact, human beings are not “designed” to manage segregation for a long time. As the Greek philosopher Aristotle reminds us, man is a “social animal,” unable to live isolated from others, since the absence of relationships removes essential conditions for the development of personal identity and the exercise of reason. Although our first instincts may be to react angrily at (and with) people who pour out onto the streets, there is a need for a more universal compassionate stance – and recognition that the very nature of the human being is in stark contrast with the situation we are experiencing.

Moreover, research shows that nourishment and movement – besides being important therapeutic expedients – are a fundamental vehicle for communicating with ourselves, others, and the world, and have an enormous influence on our biopsychological balance.

Prolonged isolation can adversely affect physical and emotional health, altering sleep and nutritional rhythms, as well as reducing opportunities for movement (Cacioppo and Hawkley, 2003). As a result, the natural channels of human expression and pleasure become depressed, with attendant impacts on mood and subjective well-being (Nardone and Speciani, 2015).

Furthermore, in accordance with current regulations, we have begun to behave “as if” other people are potentially dangerous for our health and for the health of our loved ones. This turn of events has cultivated a new universal belief based on vulnerability-to-harm, whereby proximity to fellow human-beings poses a direct threat (Nardone and Portelli, 2005). To date, more and more people are avoiding social relations, no longer by imposition, but as a choice. A decision initially moved by the fear of an invisible enemy and by the total uncertainty about what is right to do/not to do, to say/not to say, to think/not to think, derived from the information – ambiguous and conflicting – that we have received. In turn, this determines behavior that will gradually replace our old worldview and interpersonal relationships.

While the levels of environmental stress continue to rise, we are witnessing the deterioration of relationships. Rather than connecting people, restrictive measures are creating rivalries and arousing discord between people. As conveyed by the Latin phrase “*Divide et impera*” (literally divide and conquer), an authority that exerts high levels of control and division in governing a population, tends to fragment them. The magnitude and impact of fragmentation can be influenced substantially by leadership style. Grandiose leadership, for example, may create the seductive illusion of safety, with claims of invincibility and omnipotence, while providing an outlet for a range of grievances associated with inequalities and poverty through paranoia and blame of perceived “enemies.” These processes provide fuel for xenophobia and deeper divisions within society (Case and Maner, 2014; O’Reilly and Hall, 2020).

Anger and nervousness, unspoken and lasting, come back to haunt us with psychological problems.

Likewise, spending an unusual amount of time together in confined spaces – often unsuitable for the purpose itself – increases the risk of conflicts and domestic violence. China has experienced a significant rise in separations and divorces, particularly stressful events, which can act as a trigger – especially among the most sensitive – for the development of mental health problems, primarily depression.

On the other hand, prolonged social isolation characterized by reduced social connections and contact, generates deep disconnection among those who live alone or cannot rely on an adequate social network, thus increasing the likelihood that depressive symptoms will emerge. Social isolation has been linked to cognitive impairment, reduced immunity, increased risk of cardiovascular disease, and ultimately, mortality (Cohen et al., 1997; Bassuk et al., 1999; Barth et al., 2010; Heffner et al., 2011). The association between physical frailty and social isolation has been linked to heightened inflammatory activity, as indicated by increased levels of C-reactive protein and fibrinogen (Loucks et al., 2006).

Social isolation and loneliness are related concepts and often coexist – loneliness can lead to isolation, and vice versa (Shankar et al., 2011). Loneliness has been an emerging issue in society in recent years, and has been linked to depression, irritability, and preoccupation with negative self-related thoughts, alongside a 26% increase in risk of premature death. Research suggests that this has been a growing problem in industrialized countries, with approximately one-third of the population affected, and one in 12 people affected at a severe level. Further, it appears that income and socioeconomic status are no barrier to loneliness – everyone is equally at risk (Cacioppo et al., 2015; Holt-Lunstad and Smith, 2016).

Loneliness is increasingly recognized as a public health issue, especially due to the detrimental effects on health and potential for premature mortality (Grant et al., 2009; Cole et al., 2015; Murthy, 2017; Yanguas et al., 2018; Bzdok and Dunbar, 2020). Loneliness is associated with feelings of emptiness, sadness, and shame, alongside the subjective perception that one is disconnected from others. It not only can occur in the context of social isolation but can also persist beyond this and can be experienced even when others are physically present. Like social isolation, loneliness has been linked to depression (Cacioppo et al., 2006; Han and Richardson, 2010), increased cortisol levels (Edwards et al., 2010; Miller, 2011), lowered immunity (Cole et al., 2011), and clinical disease, with attendant increases in length and frequency of hospital stays (Thurston and Kubzansky, 2009; Hawker and Romero-Ortuno, 2016). Further, social isolation and loneliness may be stronger predictors of suicidality than other well-known risk factors, such as anxiety and hopelessness (Hom et al., 2017). In spite of the clear risks associated with loneliness, treatments to date based on cognitive-behavioral principles have shown poor outcomes (Masi et al., 2011). With the onset of COVID-19, enforced social isolation is likely to be exacerbating what is already a significant issue in our society (Hughes et al., 2004).

Added to this is the devastating and understandable impact of concerns related to economic problems and the loss of a loved one. During the coronavirus epidemic, we are forced to deal with death in ways unrelated to human civilization: from the thought of not being able to be with the deceased in his/her last moments of life, to the sense of guilt for the idea of having inadvertently infected the person, to the distress of not being able to properly honor him/her with a funeral ceremony, fundamental to the process of mourning – these are all factors that amplify the pain of death, increase the rates of depression, the consumption of alcohol, drugs and risky behaviors and, in the more extreme cases of suicide.

Unlike the common and ineliminable moments of crisis that characterize the existence of each of us – which, although destabilizing, represent a unique and fundamental opportunity to review personal strategies for problem management – in this period, people are experiencing impotence, vulnerability, and a feeling of loss of control over one’s life as a response to something indeterminate in time and space. This generates anguish for an uncertain future and, once again, favors the appearance of depressive symptoms – especially in those most vulnerable, including those who already suffered from mental health problems and in health workers.

Those who have been placed in quarantine and those working on the front lines to deal with the epidemic are also at risk of being stigmatized: as possible “plague-spreaders,” they are viewed with fear and suspicion.

Certainly, some will prove to be more resilient than others and will be able to count on the presence of greater personal, social, and economic resources, but we all will be affected – to varying degrees – by the impact of this revolution on our way of living and relating to each other and on our physical and psychological health.

## A Storm of Risks for Depression

The environmental stressors that characterize this particular historical moment clearly suggest the risk of a new epidemic, and this time there are signs it could be our mental health; but the national health system, once again, may not be ready to stem the effects of the epidemic.

As the reality of social isolation persists throughout and beyond the pandemic, loneliness and interpersonal disconnection will emerge, particularly for those most socially vulnerable. Psychophysical exhaustion, anxiety, fear and pain, anguish, trauma, and anger – these emotions alternate, mix, and grow in intensity to the point of overwhelm, leading to clinically significant psychological disorders, such as “reactive depression.”

While the COVID-19 crisis increases the risk of depression, depression affects the individual’s ability to solve problems, set and achieve goals, and function effectively, at work and in relationships, making recovery from the crisis even more difficult. In fact, even if it manifests in different ways, at the basis of depression there is always an attitude of renouncement. People gradually lose any form of active reactivity in the face of life’s difficulties: there is an increasing tendency to complain, let off steam, and rely completely on others in the management of themselves, all actions of delegation, therefore of renunciation. And, as described by Emile Cioran, the renunciation is nothing more than “a small daily suicide.”

Feeling safe and protected is a fundamental primary need of the human being to be able to move freely in the surrounding world, as well as the feeling of having control over the events of our own life. When all this fails, when the belief that whatever we do will not improve things begins to develop, a sense of “learned helplessness” takes hold, blocking any possibility of liberation or change.

## How to Prevent COVID-19 Depression

The anguish we experience is a normal human response to a serious crisis. Recognizing and accepting these feelings prevents them from turning into disorder.

Giving up, delegating, and complaining are all attitudes that at the beginning of a crisis can help us, but after several months can become entrenched, self-perpetuating, and end up complicating the situation, evolving as a slow drift into a depressive mindset. Recognizing these patterns immediately in one’s thought processes and behavior is the best way to move in the opposite direction and to break the vicious circle that leads to global renunciation – and that characterizes the most severe depressive forms.

This pandemic will inevitably lead to redefining our relationship styles, which will no longer be based on proximity but on distance. Physical contact will be replaced by negotiated sharing, while the digitalization of lives, already started with the advent of social media, technology, and virtual reality, will be further emphasized, thanks to medical-scientific legitimacy.

Abandoning the idea that “things will go back to normal” and facing the changes taking place with flexibility mitigates the onset of psychopathology.

The human being – by nature – is extremely flexible – facilitating adjustment to the reality that change will become the new normality (Rossi et al., 2020). In Lao Tzu’s words, “Water is fluid, soft, and yielding. But water will wear away rock, which is rigid and cannot yield. As a rule, whatever is fluid, soft, and yielding will overcome whatever is rigid and hard. This is another paradox: what is soft is strong.” But it takes time.

Specific treatment options are available for the most problematic situations, and more available than before the advent of the coronavirus, as mental health professionals – even the most resistant – are – *flexibly* – offering online support and advice.

First, however, there is a need for higher level changes: state economic support measures are crucial responses to both the economic recession and the psychological depression. Institutions must ensure that this experience is as tolerable as possible for people. Alarmist messages, such as the emphasis on the negative aspects of the pandemic (number of seriously ill people or deaths) rather than on the positive ones (number of recovered), the abuse of alarmist expressions (“death even among young people”), and stories rich in personal details about the victims, are as counterproductive as excessive references to positivity and optimism, which, on the other hand, produce a paradoxical effect: the unrealistic nature of the messages may lead to greater mistrust and perhaps dismay (“they do not tell it as it is”). Even vague or ambiguous messages (“if we are united, everything will be fine,” “be responsible,” “stay alert, control the virus”) dilute the desired effects.

Human resilience is closely linked to the depth and strength of our interpersonal connections, including our involvement in groups and communities. In contrast, loneliness appears to be one of the greatest threats to our health, survival, and well-being. In an atmosphere of uncertainty and fear, it is essential that clear and precise information is provided on the problem and on the management of the emergency. Greater cultural and economic investments will therefore have to emerge to support better and more timely prevention, treatment, and rehabilitation programs in the field of mental health, because “there is no health without mental health.”

## Data Availability Statement

The original contributions presented in the study are included in the article/supplementary material, further inquiries can be directed to the corresponding author.

## Author Contributions

GP drafted and edited the manuscript. SS critically revised the manuscript. All authors contributed to the article and approved the submitted version.

### Conflict of Interest

The authors declare that the research was conducted in the absence of any commercial or financial relationships that could be construed as a potential conflict of interest.
